# Mechanical and Microstructural Response of FDM-Printed PETG and PETG+CF to Variable Infill Architecture and Lubricant Exposure

**DOI:** 10.3390/polym18050654

**Published:** 2026-03-07

**Authors:** Lidija Rihar, Elvis Hozdić

**Affiliations:** Faculty of Mechanical Engineering, University of Novo Mesto, Na Loko 2, SI-8000 Novo Mesto, Slovenia; elvis.hozdic@fs-unm.si

**Keywords:** FDM/FFF, PETG, PETG+CF, infill pattern, infill density, tensile properties, mineral engine oil exposure, porosity quantification, optical microscopy, process–structure–property

## Abstract

Fused deposition modelling/fused filament fabrication (FDM/FFF) enables rapid manufacturing of functional polymer components; however, the reliability of printed parts remains strongly governed by internal architecture, process-induced porosity, and exposure to service fluids. This study quantifies the combined influence of (i) infill pattern (linear, triangular, hexagonal) at 30% density, (ii) infill density (30%, 60%, 100%) for linear infill, and (iii) short-term lubricant exposure on the tensile and microstructural response of FDM-printed polyethylene terephthalate glycol-modified (PETG) and short-carbon-fibre-reinforced PETG (PETG+CF). Specimens were printed following ISO 527-2 and tensile-tested at 5 mm/min. Microstructural analysis coupled quantitative porosity with mechanical response, Young’s Modulus, and strain-to-break. At 30% density, PETG with hexagonal infill achieved the highest tensile strength (18.54 ± 0.67 MPa), exceeding linear (16.99 ± 0.52 MPa) and triangular (14.15 ± 0.70 MPa) patterns, while triangular and linear patterns exhibited higher Young’s Modulus, indicating topology-driven decoupling of stiffness and strength. Increasing linear infill density raised strength to 31.35 ± 0.33 MPa (PETG) and 38.90 ± 0.28 MPa (PETG+CF) at 100%, consistent with reduced porosity. Seven-day immersion in SAE 15W-40 mineral engine oil reduced PETG strength by ~17% while increasing deformation to failure, whereas PETG+CF showed only minor changes. Overall, the results demonstrate that architecture-aware design, supported by quantitative porosity descriptors, is essential for ensuring the reliable mechanical performance of FDM/FFF-printed PETG-based components exposed to service fluids.

## 1. Introduction

Polymer-fused deposition modelling/fused filament fabrication (FDM/FFF) is increasingly used beyond prototyping, which makes it essential to interpret tensile behaviour in a way that is comparable and design-relevant. ISO 527-2 [1] is therefore commonly adopted for specimen geometry and test conditions, yet the measured response of printed parts is strongly shaped by process-generated mesostructure—especially imperfect inter-road/interlayer fusion and voids that act as stress concentrators. A practical way to link these defects to macroscopic properties is optical microscopy combined with reproducible image-analysis workflows (e.g., Fiji/ImageJ and global thresholding such as Otsu), which supports a process–structure–property interpretation of FDM/FFF components [1,2,3,4].

Polyethylene terephthalate glycol-modified (PETG) has become a popular FDM/FFF feedstock because it offers a useful balance of printability and mechanical performance; however, its tensile response is highly sensitive to processing-induced morphology. Prior studies on FDM-printed PETG show that parameters such as nozzle temperature and printing speed control interlayer diffusion and void formation, which in turn shifts strength, stiffness, and ductility [5,6,7,8]. For reinforced systems, broader reviews similarly emphasize that the benefits of reinforcement can be limited by process defects and imperfect bonding when consolidation is insufficient [9].

PETG is now reported in diverse application-oriented contexts (engineering, architectural and biomechanical uses), reinforcing the need for experimentally grounded, architecture-aware property data rather than reliance on nominal material values [10,11,12,13]. Comparative and optimization studies further demonstrate that printing parameters can change both thermal and mechanical response, motivating reproducible workflows that support parameter selection and modelling for design [14,15,16]. In addition, PETG properties may evolve with storage or ageing (e.g., hydrolytic degradation), and cross-polymer reviews (PLA/ABS/TPU/PETG) consistently stress that performance should be interpreted in a process- and defect-dependent context rather than as an intrinsic constant [17,18,19,20,21,22,23].

In practice, internal architecture is deliberately adjusted to reduce mass and build time while meeting load requirements; infill pattern and infill density are two of the most widely used “design knobs.” For PETG, infill topology can change load paths and stress concentration sites even at the same density, while densification generally raises strength by increasing effective load-bearing area and reducing void content [24,25]. Pattern-dependent behaviour has also been linked to surface quality and post-processing/annealing effects, and design-of-experiment studies highlight that topology effects are often statistically detectable but difficult to generalize without defect descriptors [26,27,28,29,30,31,32]. Although non-destructive techniques for porosity assessment exist, microscopy-based quantitative workflows remain widely accessible for systematic comparisons across architectures [33].

Short carbon fibre (CF) reinforcement is frequently used to improve stiffness and functional performance in FDM polymers, yet the realized benefit depends on fibre–matrix adhesion, fibre orientation along deposited roads, and the same void/interlayer defects that govern neat polymers. Reviews on carbon-fibre-reinforced printed composites and continuous-fibre strategies underline the strong coupling between printing strategy, consolidation quality and mechanical response [34,35,36]. For PETG-based composites, studies report changes in printability, mechanical response and tribological/fatigue behaviour, again pointing to the need to interpret reinforcement effects together with architecture-controlled defect states [37,38,39,40,41,42,43,44,45].

For end-use adoption, FDM/FFF polymers must also retain performance under environmental and chemical exposure. Reviews and accelerated ageing studies show that radiation, moisture, disinfectants and other service conditions can shift the mechanical behaviour of printed polymers in a material-specific way [46,47,48,49,50,51,52,53,54,55,56]. In machinery-related applications, contact with lubricants is a common service scenario; prior work indicates that SAE 15W-40 mineral engine oil can measurably change mechanical parameters of FDM polymers (including PETG-based systems), which motivates controlled lubricant-exposure testing for architecture-optimized components [57,58].

Despite extensive research on individual process parameters, architecture settings and environmental exposure, direct comparison across studies is often limited by differences in specimen geometry, printing conditions, and test protocols. Many reports vary infill topology, infill density, or exposure conditions in isolation, which complicates translation into robust design rules for lightweight load-bearing components.

For PETG and short-carbon-fibre-reinforced PETG (PETG+CF), there is still limited protocol-controlled evidence that (i) compares multiple infill topologies at a fixed low density, (ii) quantifies densification within a single topology, and (iii) evaluates the lubricant exposure representative of service contact, while simultaneously linking tensile response to quantitative porosity descriptors within one unified framework.

Accordingly, the aim of this study is to establish a unified process–structure–property dataset for PETG and PETG+CF by combining ISO-based tensile testing with microscopy-assisted, quantitative porosity metrics under controlled architectural variation.

Specifically, we investigate the following: (a) infill pattern (linear, triangular, hexagonal) at 30% density, (b) infill density (30%, 60%, 100%) for linear infill, and (c) the effect of a short-term lubricant exposure (7-day immersion in SAE 15W-40 mineral engine oil) for a representative low-density configuration. Mechanical outcomes (tensile strength, nominal Young’s modulus and strain at break) are interpreted together with microscopy-derived defect descriptors.

The workflow is intentionally protocol-controlled and is designed as a continuation of a previously published unified methodology that combined infill-architecture variation, lubricant exposure, ISO 527-2 tensile testing and microscopy-based porosity quantification for PLA and PLA+CF [59]. By applying the same framework to PETG and PETG+CF feedstocks, the present study enables cross-material comparison while keeping printing, testing and image-analysis procedures consistent.

To support a structured interpretation of the results, we tested the following hypotheses: (H1) at low infill density, topology controls dominant load paths and the distribution of stress concentrators, which can decouple apparent stiffness from strength and ductility; (H2) increasing infill density reduces voided area and increases tensile strength in both PETG and PETG+CF; and (H3) mineral engine oil exposure shifts the strength–ductility balance, with the magnitude of change depending on reinforcement and interfacial integrity.

## 2. Materials and Methods

Two PETG-based filaments were selected to enable a controlled comparison between an unreinforced thermoplastic and a short-fibre composite manufactured and tested under the same conditions. Commercial 1.75 mm PETG and PETG+CF filaments (FlashForge) supplied by Zhejiang FlashForge 3D Technology Co., Ltd. (Jinhua, China) were used as received [60].

According to the supplier, the PETG+CF grade (PETGCF10) contains 10 wt% short carbon fibres. The technical data sheet does not report fibre diameter, the post-compounding fibre-length distribution, or the sizing chemistry because these fibre-level details can influence reinforcement efficiency; reinforcement effects are interpreted here at the printed-part level within the controlled process–structure–property framework.

To isolate material effects, identical printing, tensile testing and microscopy/porosity quantification procedures were applied to both materials. Supplier-declared filament specifications and recommended processing windows are summarized in Table 1 [60], while nominal filament mechanical properties (Table 2) are provided only as feedstock descriptors and are not assumed to represent printed-part properties, which are governed by bonding quality, porosity and architecture.

To limit moisture-related variability in extrusion behaviour and interfacial bonding, both filaments were dried at 60 °C for 8 h in a ventilated laboratory oven and stored in sealed containers with desiccant until printing.

**Figure 1:**
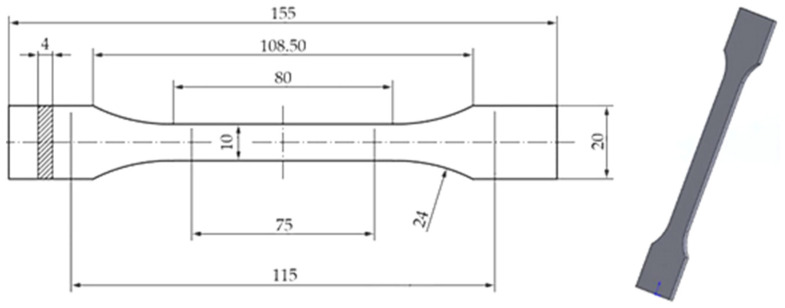
SolidWorks-generated model of the standardized tensile specimen geometry (ISO 527-2:2012) [1].

Tensile specimens were designed according to ISO 527-2:2012 [1]. The dog-bone geometry (overall length 155 mm; gauge length 80 mm; gauge width 10 mm; thickness 4 mm; end width 20 mm) was modelled in SolidWorks 2023 (Dassault Systèmes, Bouguenais, France) and sliced in FlashPrint 5. Specimens were printed using an Adventurer 4 Series FDM/FFF printer (FlashForge) equipped with a 0.4 mm hardened-steel nozzle (by Zhejiang FlashForge 3D Technology Co., Ltd. [Jinhua, China]). The standardized specimen geometry is shown in Figure 1.

The experimental plan varied three factors while keeping all other printing conditions constant: (i) infill pattern at a fixed low density (30%), (ii) infill density (30%, 60%, 100%) for a selected pattern (linear), and (iii) lubricant exposure representative of service contact. The 30% condition was chosen as a lightweight architecture where topology-driven load paths and void architecture are expected to strongly influence tensile response.

**Figure 2:**
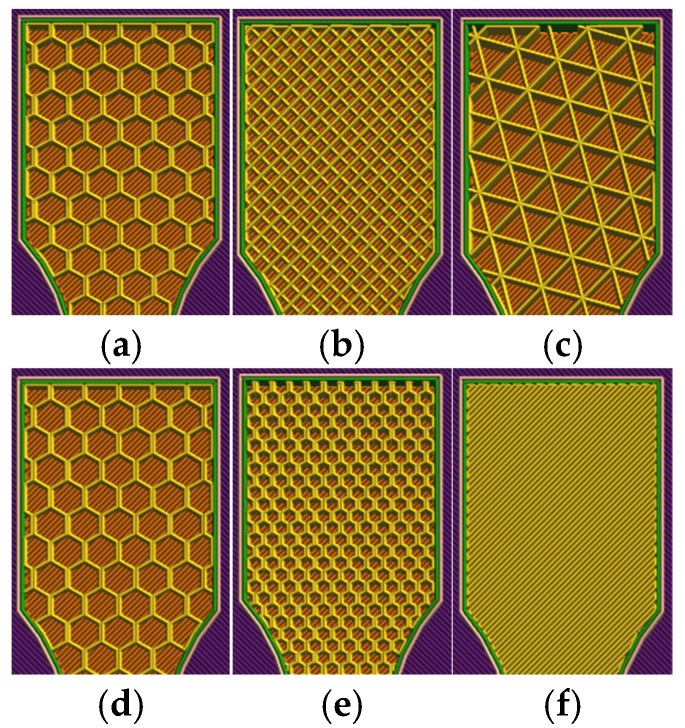
Representative slicer-generated views of internal architectures used in this study: (**a**–**c**) infill pattern series at 30% infill density (linear, triangular, hexagonal); (**d**–**f**) infill density series for the selected pattern (linear) at 30%, 60%, and 100%.

Three infill topologies—linear (rectilinear), triangular and hexagonal (honeycomb)—were first evaluated at 30% infill density, see Figure 2. Consolidation effects were then quantified by increasing the density of the linear pattern to 60% and 100%. Finally, lubricant exposure was examined by comparing unexposed and oil-immersed specimens for the 30% hexagonal configuration. Throughout the paper, suffix “A” denotes tensile-tested specimens and suffix “B” denotes unfractured companion specimens prepared for microscopy. PETG configurations were coded as V13 (hex 30%), V14 (tri 30%), V15 (lin 30%), V16 (lin 60%), V17 (lin 100%) and V18 (hex 30% after oil exposure); PETG+CF configurations were V19, V20, V21, V22, V23 and V24, respectively. For linear infill, the raster direction was set to 0° relative to the tensile axis.

All other slicing/printing parameters were held constant and selected within the supplier-recommended ranges (Table 1). Only infill pattern, infill density and oil exposure were varied. Nozzle and bed temperatures were material-specific but fixed for each material across its full test matrix.

After printing, specimens were conditioned for 48 h at 23 ± 2 °C and 50 ± 5% RH prior to mechanical testing. Lubricant exposure was applied only to the 30% hexagonal configuration (PETG: V13 vs. V18; PETG+CF: V19 vs. V24) by full immersion in SAE 15W-40 mineral engine oil for 7 days in sealed glass containers at 25 ± 2 °C. After immersion, specimens were removed, gently wiped to remove surface oil, and conditioned at room temperature for 24 h before testing.

This immersion protocol is intended as a short-duration screening test under static conditions. To keep the study protocol-controlled and avoid excessive parameter proliferation, only one representative low-density architecture was exposed, and no gravimetric uptake, penetration depth or chemical analyses (e.g., FTIR) were performed.

Tensile tests were performed in accordance with ISO 527-2:2012 [1] using a Shimadzu AGS-X universal testing machine (Kyoto, Japan) with a 10 kN load cell and an integrated TrapeziumX software (V 3.6). Tests were conducted at 23 ± 2 °C and 50 ± 5% RH using a constant crosshead speed of 5 mm/min. Strain was measured with an extensometer (instrument accuracy ±0.5%, per manufacturer specification). From each curve, maximum force (F), maximum displacement, tensile strength, Young’s modulus and nominal strain at break were extracted. For infill densities below 100%, stresses were calculated using the nominal (gross) cross-sectional area (10 mm × 4 mm) to preserve direct comparability across architectures, consistent with the adopted process–structure–property comparison approach [59].

(1) $$\sigma_{m}=\frac{F_{max}}{A_{0}}$$

where $F_{max}$ is the maximum force and $A_{0}$ is the nominal gross cross-sectional area of the gauge Section (10 mm × 4 mm).

Young’s modulus was obtained from the slope of the initial linear portion of the nominal stress–strain response using a fixed elastic strain window of 0.05–0.25% after toe-region removal. Nominal strain at break was taken as the extensometer strain at fracture.

Because the same gross cross-section was used for all infill levels, the reported modulus represents an apparent structural stiffness rather than an intrinsic solid-material modulus—particularly at low infill densities where early deformation includes filament bending and contact closure. The modulus is therefore used for relative comparisons across configurations rather than as a material constant.

For each condition, three specimens were tested (n = 3). Results are reported as mean ± SD and as 95% confidence intervals (CIs) of the mean, calculated using a t-interval with n = 3 (df = 2): CI = $\overset{\text{-}}{x}$ ± t0.975,2 · (s/√n).

One-way ANOVA (α = 0.05) followed by Tukey’s HSD post hoc test was used to evaluate infill-pattern and infill-density effects on tensile strength. For two-group comparisons (oil exposure), Welch’s *t*-test was applied. Analyses were performed in Python (v 3.11; SciPy and statsmodels).

Given the exploratory nature of the matrix and the limited replication (n = 3), statistical testing was used as supporting evidence alongside effect magnitudes, confidence intervals and consistency with microstructural indicators, rather than as the sole basis for conclusions.

**Figure 3:**
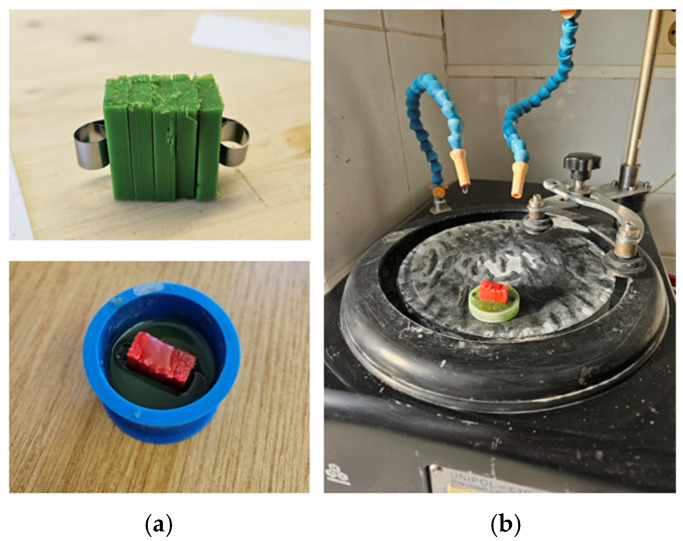
Sample preparation and surface finishing: (**a**) preparation and sectioning of specimens prior to embedding; (**b**) progressive surface grinding and polishing of mounted samples (protocol consistent with the continuation workflow) [59].

Microstructural characterization was performed to relate tensile behaviour to internal morphology and void architecture. After tensile testing, fracture surfaces were documented macroscopically using a VEVOR digital microscope. For cross-section microscopy, unfractured companion specimens (“B”) were sectioned from the gauge region into ~15 mm segments, embedded in epoxy, and ground/polished using SiC papers (Struers, Copenhagen, Denmark; P240, P800, P1200 and P2500) followed by alumina polishing (~0.05 µm), consistent with the continuation protocol [59]. The sample preparation and surface finishing workflow is documented in Figure 3.

Optical microscopy was carried out using a Zeiss Axio Imager A1m microscope (Carl Zeiss Microscopy, LLC, White Plains, NY, USA) with AxioVision software (Rel. 4.8). Images were calibrated with a micrometric scale (10 µm per division). The analyzed field of view (FOV) was approximately 2.5 mm × 1.9 mm; five FOVs per “B” specimen were analyzed to capture within-sample variability. Where full cross-sections were needed for presentation, images were stitched and reported with a 500 µm scale bar.

Quantitative porosity analysis was performed in Fiji (ImageJ distribution, Version 2.14.0) [2] using a single fixed segmentation workflow for all conditions. Images were first calibrated and background-normalized, then smoothed using Gaussian filtering (σ = 1.0) to reduce illumination gradients and improve segmentation robustness, consistent with the continuation framework. Pores were segmented using global Otsu thresholding [3], followed by morphological opening and closing (disc structuring element, radius = 2 px). A 20-pixel guard band was applied to exclude edge-intersecting pores and the analyzed area was corrected accordingly. Only features with equivalent areas between 10 and 100,000 µm^2^ were retained for quantification.

To preserve methodological continuity with our earlier PLA/PLA+CF study [59], the same thresholding strategy and key processing parameters were used. The resulting metrics are therefore intended for consistent, relative comparison under identical imaging and segmentation settings rather than as absolute porosity values; the workflow was not independently re-tuned for PETG.

The 2D areal porosity fraction $\phi_{A}$ was computed as follows:

(2) $$\phi_{A}=\frac{A_{pore}}{A_{analyzed}}$$

In addition to the areal porosity fraction ($\phi_{A}$), we extracted the number of pores ($N_{p}$), total pore area ($\sum A_{p}$) and mean pore area ($\bar{A_{p}}$) for each FOV, and then aggregated these descriptors per specimen.

Image acquisition and processing settings were held constant across all specimens to minimize user-induced bias.

With this consistency, φA and the associated descriptors provide a reproducible basis for comparative ranking of topology-, density- and exposure-driven changes in voided area and inter-road/interlayer gaps within the controlled framework [2,3,59].

The porosity descriptors reported here are based on 2D polished cross-sections and thus represent areal metrics rather than true volumetric porosity. In anisotropic FDM/FFF structures, 2D measurements cannot capture 3D connectivity or tortuosity of pore networks.

Nevertheless, when applied with identical preparation, imaging and segmentation settings, 2D metrics remain suitable for tracking relative trends and correlating void morphology with tensile response at the scale relevant for fracture initiation. Volumetric techniques (e.g., micro-CT) are identified as future work.

For PETG+CF, reflected-light microscopy was used primarily to quantify porosity and bonding integrity; detailed fibre metrics (e.g., orientation and length distribution) were not quantified in this study. Reinforcement effects are therefore interpreted from the combined mechanical response and porosity descriptors obtained under identical process conditions.

## 3. Results

This section presents the tensile response and corresponding microstructural descriptors of FDM/FFF-printed PETG and PETG+CF as a function of (i) infill geometry at constant infill density (30%), (ii) infill density for a linear infill architecture (30%, 60%, 100%), and (iii) 7-day mineral engine oil exposure for the 30% hexagonal configuration. Mechanical outcomes are reported as maximum force ($F_{max}$), measured tensile strength ($\sigma_{m}$), measured Young’s modulus ($E_{m}$), nominal strain at break ($\varepsilon_{b}$), and maximum displacement (${\Delta L}_{max}$). Unless otherwise stated, each condition comprises n = 3 tensile specimens; accordingly, mean values are interpreted together with scatter (SD) and internal consistency across independent indicators, including mechanical response, fracture morphology, and image-based porosity descriptors. Owing to the limited sample size, formal statistical testing is applied only as supportive evidence for the directional confirmation of pronounced effects, while the primary emphasis is placed on the magnitude, robustness, and consistency of observed trends.

To support this analysis, one-way analysis of variance (ANOVA) was performed for selected datasets, followed by Tukey’s post hoc test where applicable. Statistical results are therefore reported in a descriptive manner, with *p*-values considered indicative rather than definitive, and interpreted in conjunction with effect sizes and confidence-interval overlap.

For traceability between mechanical testing and microscopy, specimen codes are used consistently throughout this section. For PETG: V13A/V13B (hexagonal 30%), V14A/V14B (triangular 30%), V15A/V15B (linear 30%), V16A/V16B (linear 60%), V17A/V17B (linear 100%), and V18A/V18B (hexagonal 30% after 7-day oil exposure). For PETG+CF: V19A/V19B (hexagonal 30%), V20A/V20B (triangular 30%), V21A/V21B (linear 30%), V22A/V22B (linear 60%), V23A/V23B (linear 100%), and V24A/V24B (hexagonal 30% after 7-day oil exposure).

**Figure 4:**
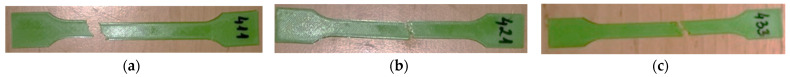
Tensile test specimens manufactured from PETG using three different internal architectures: (**a**) hexagonal (V13A/PETG_411), (**b**) triangular (V14A/PETG_421), and (**c**) linear (V15A/PETG_433), at an infill density of 30%.

**Figure 5:**
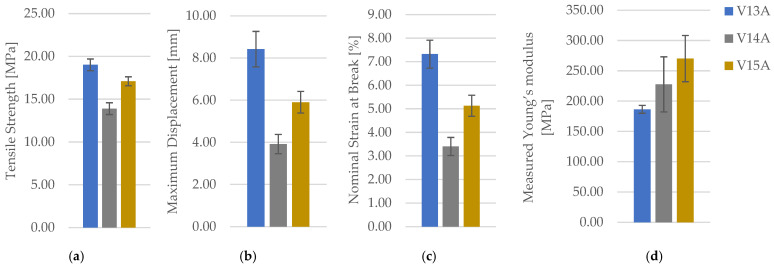
Tensile properties of representative PETG specimens printed with different infill geometries, presented as (**a**) tensile strength, (**b**) maximum displacement, (**c**) nominal strain at break, and (**d**) measured Young’s modulus.

Representative PETG tensile specimens manufactured with hexagonal, triangular, and linear architectures at 30% infill density are shown in Figure 4. The complete tensile dataset for these PETG pattern conditions is provided in Table 3 (for clarity and readability; only averaged values (mean ± SD) are reported in the main text, while full individual datasets are provided in the Supplementary Materials Table S1); the representative force–displacement curves and consolidated property comparisons are presented in Supplementary Materials (Figure S1), with graphical summaries in Figure 5.

At constant 30% density, PETG exhibits a clear architecture-driven shift in global response: hexagonal infill delivers the highest mean, triangular infill yields the lowest and the lowest deformation capacity, and linear infill remains intermediate in strength while exhibiting a stiffer initial response than hexagonal. Importantly, the ranking of differs from the ranking of, indicating that at low density the architecture can decouple measured stiffness from measured strength. In practical terms, the hexagonal network provides the most favourable strength–ductility balance among the tested patterns (highest and), whereas triangular infill produces the most limited deformation to failure. The pattern effects are large relative to within-group scatter; accordingly, one-way comparisons (ANOVA/Kruskal–Wallis, used descriptively) indicate that differences and deformation measures are resolved at the chosen α-level, while force-based differences are directionally consistent but more sensitive to variability inherent to n = 3. Supportive one-way ANOVA confirms a significant effect of infill pattern on PETG tensile strength (F(2,6) = 36.81, *p* = 4.28 × 10^−4^). Tukey HSD indicates that triangular infill yields significantly lower tensile strength than both hexagonal (*p* = 4.0 × 10^−4^) and linear (*p* = 3.8 × 10^−3^), whereas the hexagonal–linear difference is marginal at α = 0.05 (*p* = 5.46 × 10^−2^).$\;\sigma_{m}F_{max}\sigma_{m}\sigma_{m}E_{m}\varepsilon_{b}{\Delta L}_{max}\sigma_{m}$.

**Figure 6:**
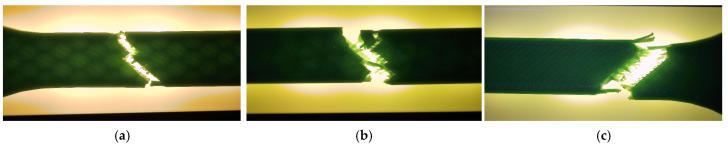
Fracture surfaces of representative PETG specimens after tensile testing: (**a**) hexagonal structure (V13A/PETG_411), (**b**) triangular structure (V14A/PETG_421), (**c**) linear structure (V15A/PETG_433).

**Figure 7:**
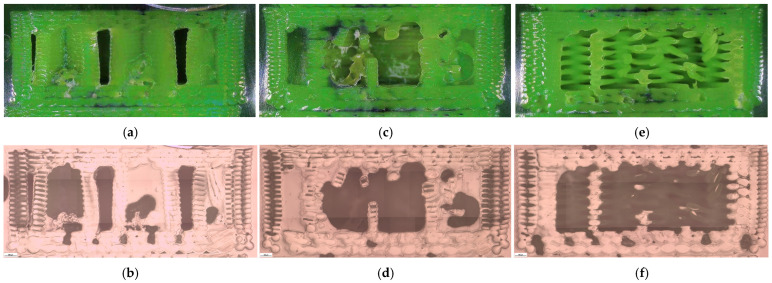
Optical microscopy images of unfractured PETG specimens illustrating the internal infill architectures: (**a**,**b**) hexagonal (V13B), (**c**,**d**) triangular (V14B), and (**e**,**f**) linear (V15B).

**Figure 8:**
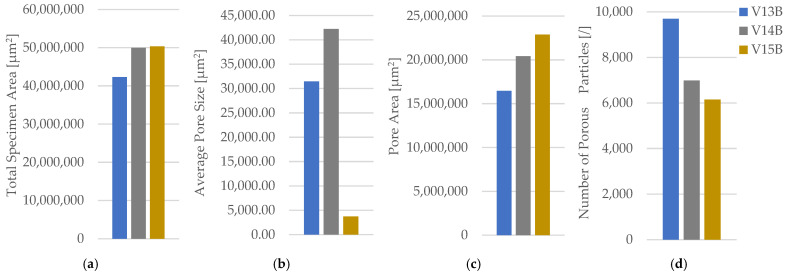
Quantitative microstructural analysis of representative PETG specimens with different infill geometries obtained using Fiji software (ImageJ distribution, Version 2.14.0; LOCI, University of Wisconsin–Madison, WI, USA): (**a**) total specimen area, (**b**) average pore size, (**c**) pore area, and (**d**) number of porous particles.

The corresponding fracture appearances support the mechanical ranking. Figure 6 shows that hexagonal PETG tends to fail with a more distributed fracture region and visibly larger global deformation, while triangular and linear conditions more often exhibit sharper separations and localized filament/interface features. To connect the macroscopic response to internal morphology, unfractured companion specimens were examined by optical microscopy. Representative micrographs for the three PETG infill patterns are provided in Figure 7 and the corresponding Fiji-derived pore descriptors are summarized in Table 4 with graphical summaries in Figure 8.

The micrographs confirm geometry-dependent void topology and inter-road gap distribution. When expressed as an area pore fraction ($A_{pores}/A_{total}$), porosity increases from hexagonal to triangular to linear, consistent with the observed reduction in strength from hexagonal to triangular and the intermediate performance of linear infill. Beyond pore fraction, the pattern-dependent pore size distribution is also relevant: linear infill exhibits larger characteristic void regions between raster lines, while hexagonal infill shows a more distributed void morphology, which is compatible with more stable deformation before catastrophic separation.

From a design-oriented comparative perspective, these results indicate that at low infill density (30%) infill topology represents a first-order design variable rather than a secondary geometric choice. For PETG-based systems, the hexagonal architecture provides the most favourable balance between tensile strength and ductility, whereas alternative topologies introduce defect architectures that promote earlier failure initiation. This highlights that topology selection at low density must be treated as a material-specific design decision rather than a universally transferable parameter.

**Figure 9:**
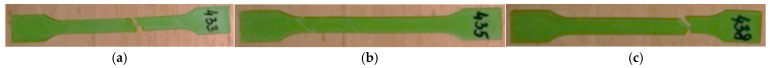
Representative tensile test specimens produced from PETG with three different infill densities: (**a**) 30% (V15A/PETG_433), (**b**) 60% (V16A/PETG_435), and (**c**) 100% (V17A/PETG_438).

**Figure 10:**
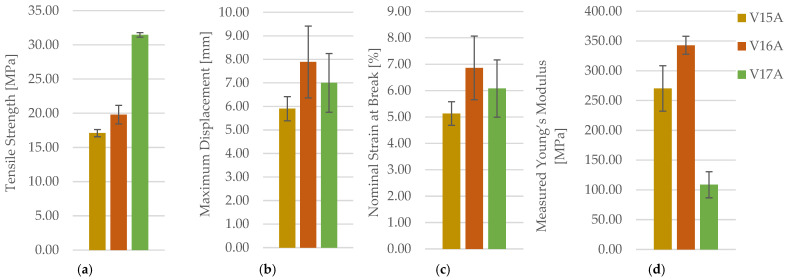
Tensile behaviour of representative PETG specimens manufactured with varying infill densities, presented in terms of (**a**) tensile strength, (**b**) maximum displacement (**c**) nominal strain at break, and (**d**) measured Young’s modulus.

The effect of infill density was then evaluated using linear infill at 30%, 60%, and 100% for PETG. Representative post-test specimens are shown in Figure 9. The full mechanical dataset is reported in Table 5 (for clarity and readability, only averaged values (mean ± SD) are reported in the main text, while full individual datasets are provided in the Supplementary Materials Table S2), with representative force–displacement curves in Supplementary Materials (Figure S2) and consolidated comparisons in Figure 10.

Increasing infill density produces a strong strengthening response, evidenced by a monotonic increase in and a corresponding rise in; the force–displacement curves shift upward systematically with density, indicating that densification increases the effective load-bearing continuity of the structure. Deformation metrics show a more nuanced response: intermediate density (60%) exhibits the highest mean deformation capacity (and), whereas 100% infill maximizes peak load and strength but does not necessarily maximize ductility. For, PETG shows an increase from 30% to 60% but a lower mean at 100% under the applied modulus-extraction protocol. Given the small sample size and the known sensitivity of Young’s Modulus in FDM/FFF specimens to early compliance, toe-region correction, and fitting-window selection, this non-monotonic trend is interpreted conservatively: the density effect on strength and load capacity is robust and large, while stiffness trends are more method-sensitive and require cautious interpretation. One-way ANOVA confirms a highly significant effect of infill density on PETG tensile strength (F(2,6) = 259.76, *p* = 1.49 × 10^−6^). Tukey HSD indicates that all density levels differ significantly (30% vs. 60%: *p* = 4.74 × 10^−2^; 60% vs. 100%: *p* < 1 × 10^−3^; 30% vs. 100%: *p* < 1 × 10^−3^).${\;\sigma }_{m}F_{max}\varepsilon_{b}{\Delta L}_{max}E_{m}E_{m}$.

The non-monotonic trends observed in the measured Young’s modulus, particularly at lower infill densities, should be interpreted with caution. In FDM/FFF-printed cellular structures, the apparent elastic modulus extracted from tensile tests is highly sensitive to early-stage compliance effects, including toe-region behaviour, local filament bending, inter-road contact closure, and the selected strain window for linear fitting. Although a fixed elastic strain range was applied consistently across all specimens, small variations in internal architecture and local consolidation can lead to measurable differences in the initial slope of the stress–strain curve. For this reason, tensile strength and force-based metrics are considered more robust indicators of load-bearing capability in low-density FDM structures, while Young’s modulus is treated as a secondary descriptor that supports, but does not dominate, the interpretation of mechanical performance.

**Figure 11:**
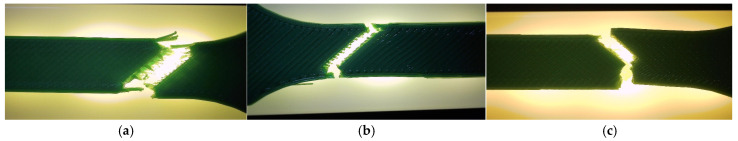
Fracture surfaces of representative PETG specimens after tensile testing: (**a**) 30% (V15A/PETG_433), (**b**) 60% (V16A/PETG_435), and (**c**) 100% (V17A/PETG_438).

**Figure 12:**
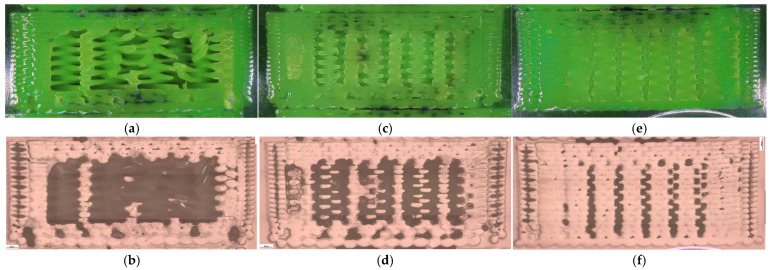
Optical micrographs of PETG specimens with different infill densities: (**a**,**b**) 30% (V15B/PETG_433), (**c**,**d**) 60% (V16B/PETG_435), and (**e**,**f**) 100% (V17B/PETG_438).

**Figure 13:**
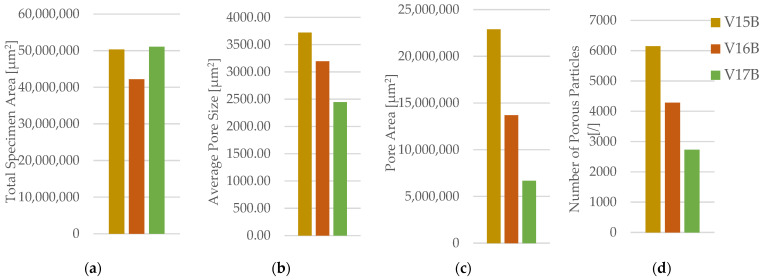
Quantitative microstructural analysis of representative PETG specimens with different infill densities obtained using Fiji software: (**a**) total specimen area, (**b**) average pore size, (**c**) pore area, and (**d**) number of porous particles.

Fracture surfaces for the PETG density series are shown in Figure 11. At higher densities, the fracture region appears more consolidated and less dominated by large void-controlled tearing, consistent with increased $\sigma_{m}$ Microstructural evidence confirms this densification-driven consolidation. Representative cross-sections are shown in Figure 12, and quantitative pore descriptors are reported in Table 6 and summarized in Figure 13.

The areal pore fraction decreases monotonically with density, and total pore area reduces markedly, providing a mechanistic basis for the monotonic strength increase. The combined mechanical–microstructural dataset therefore indicates that, for PETG under the fixed processing conditions used here, infill density is the most effective lever for increasing $\sigma_{m}$, primarily via void reduction and improved continuity of the load-bearing cross-section.

Across all investigated materials and configurations, infill density exhibits a consistent and monotonic influence on tensile strength, underscoring densification as the most robust and material-agnostic design lever for strength enhancement in FDM/FFF components. In contrast to infill topology, whose effect is pronounced primarily at low densities, increased consolidation improves load-bearing continuity irrespective of polymer chemistry or reinforcement, providing a clear prioritization rule for strength-driven design.

**Figure 14:**
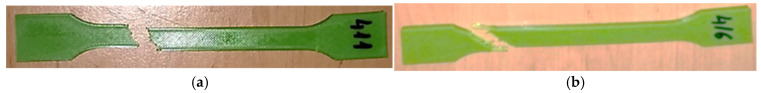
PETG tensile specimens printed with hexagonal infill (30% density) after tensile testing: (**a**) unexposed sample (V13A/PETG_411); (**b**) sample after 7 days of exposure to mineral engine oil (V18A/PETG_416).

**Figure 15:**
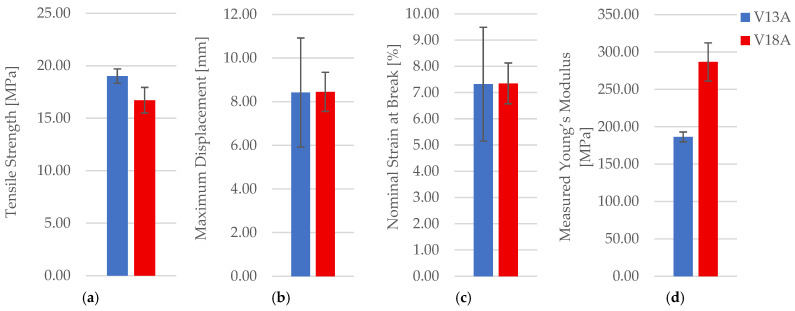
Comparison of tensile properties of representative PETG specimens before and after 7-day exposure to mineral engine oil: (**a**) tensile strength, (**b**) maximum displacement, (**c**) nominal strain at break, and (**d**) measured Young’s modulus.

The influence of mineral engine oil exposure was assessed for PETG using the 30% hexagonal configuration (unexposed V13 vs. exposed V18). Representative post-test specimens are shown in Figure 14. Mechanical results are reported in Table 7, with representative force–displacement curves in Supplementary Materials (Figure S3) and property comparisons in Figure 15. A Welch *t*-test indicates a statistically significant decrease in PETG tensile strength after oil exposure (t = 4.13, *p* = 2.21 × 10^−2^; Cohen’s d = 3.37), supporting the observed mean shift despite n = 3.

**Figure 16:**
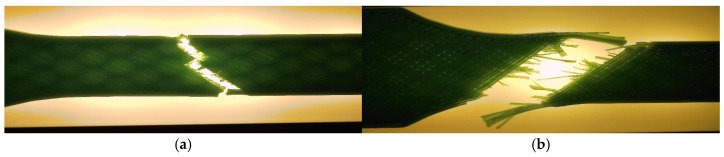
Fracture surfaces of representative PETG specimens after tensile testing: (**a**) V13A—unexposed (PETG_411), brittle fracture with clean separation; (**b**) V18A—after 7 days in mineral oil (PETG_416), showing ductile failure and localized plastic deformation.

**Figure 17:**
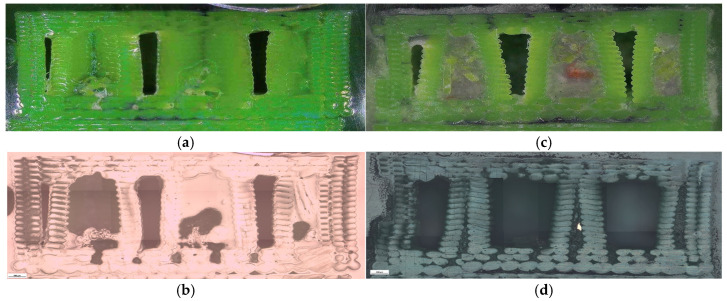
Optical micrographs of unfractured PETG specimens with hexagonal infill (30%): (**a**,**b**) unexposed (V13B/PETG_411); (**c**,**d**) after 7 days of mineral oil exposure (V18B/PETG_416).

**Figure 18:**
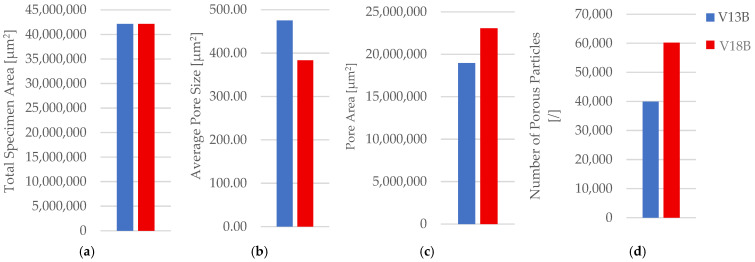
Quantitative microstructural analysis of representative PETG specimens before and after mineral oil exposure: (**a**) total specimen area, (**b**) average pore size, (**c**) pore area, and (**d**) number of porous particles.

Oil exposure reduces $\sigma_{m}$ and $F_{max}$ by a clear margin (approximately a 17% mean strength decrease), while deformation measures show small increases. With *n* = 3, these shifts are interpreted as a robust directional trend (large relative magnitude for $\sigma_{m}$), while secondary changes in $\varepsilon_{b}$ and ΔL_max remain more uncertain due to scatter. Figure 16 provides qualitative context, showing a more deformation-assisted fracture appearance in the exposed case. Microstructurally, unfractured specimens are shown in Figure 17, while pore descriptors are summarized in Table 8 and visualized in Figure 18.

After exposure, the areal pore fraction increases substantially and pore count rises, while average pore size decreases, indicating a shift towards a more fragmented void morphology (and/or increased segmentation sensitivity due to contrast changes). Taken together with the mechanical decrease in $\sigma_{m}$, the data support that oil exposure measurably alters the effective integrity of the low-density PETG architecture under the present conditions.

**Figure 19:**
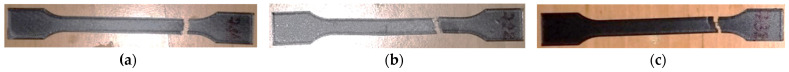
Representative PETG+CF tensile specimens printed with different infill geometries: (**a**) hexagonal (V19A/PETG+CF_711), (**b**) triangular (V20A/PETG+CF_722), and (**c**) linear (V21A/PETG+CF_732), all with 30% infill density.

**Figure 20:**
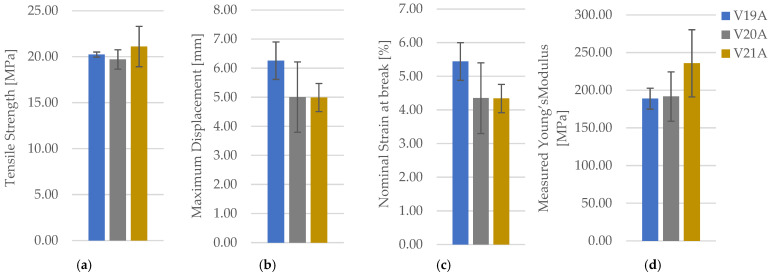
Comparison of tensile properties of representative PETG+CF specimens with different infill geometries: (**a**) tensile strength, (**b**) maximum displacement, (**c**) nominal strain at break, and (**d**) measured Young’s modulus.

For PETG+CF at 30% density, representative specimens printed with hexagonal, triangular, and linear infill patterns are shown in Figure 19. The full mechanical dataset is provided in Table 9 (for clarity and readability, only averaged values (mean ± SD) are reported in the main text, while full individual datasets are provided in the Supplementary Materials Table S4) with representative force–displacement curves in Supplementary Materials (Figure S4) and consolidated comparisons in Figure 20.

**Figure 21:**
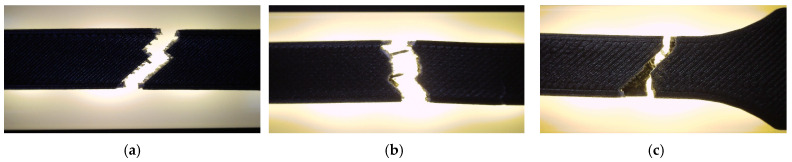
Fracture surfaces of representative PETG+CF specimens after tensile testing: (**a**) V19A—hexagonal structure (PETG+CF_711); (**b**) V20A—triangular structure (PETG+CF_722); (**c**) V21A—linear structure (PETG+CF_732). The fracture morphology reflects the interplay between infill topology and fibre–matrix adhesion.

**Figure 22:**
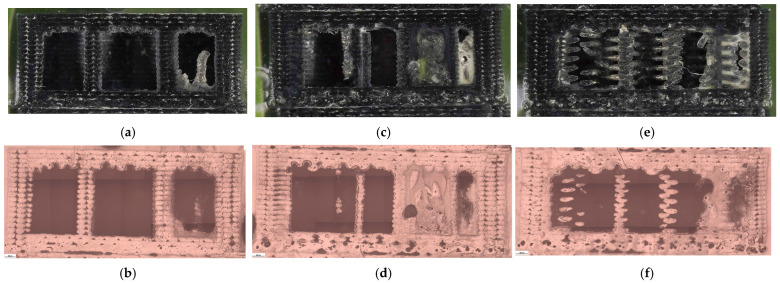
Optical micrographs of unfractured representative PETG+CF specimens showing internal infill morphology: (**a**,**b**) hexagonal (V19B/PETG_711), (**c**,**d**) triangular (V20B/PETG_722), and (**e**,**f**) linear (V21B/PETG_732) structures.

**Figure 23:**
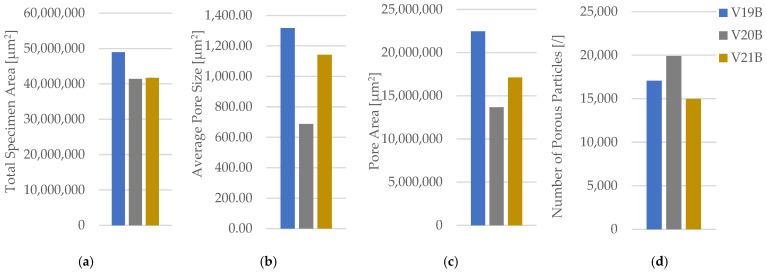
Quantitative microstructural analysis of representative PETG+CF specimens with different infill geometries obtained using Fiji software: (**a**) total specimen area, (**b**) average pore size, (**c**) pore area, and (**d**) number of porous particles.

Compared with neat PETG, PETG+CF exhibits only a modest separation in between patterns at 30% density, and the linear pattern shows the largest scatter among the three. With n = 3 per condition, descriptive ANOVA/Kruskal–Walli’s summaries indicate that pattern-to-pattern differences are not consistently resolved for and deformation metrics, implying that any true pattern sensitivity in PETG+CF at low density is smaller than, or comparable to, the between-specimen variability captured here. Fracture surfaces in Figure 21 show filament-scale texture consistent with interface-controlled damage contributions, but no distinctly different qualitative fracture mode is isolated for a single pattern at this density. Optical micrographs for PETG+CF infill patterns are provided in Figure 22. Quantitative pore metrics are reported in Table 10 and summarized in Figure 23. Supportive one-way ANOVA does not resolve a statistically significant infill-pattern effect on PETG+CF tensile strength at 30% density (F(2,6) = 0.554, *p* = 0.601), consistent with the observed overlap of scatter. $\sigma_{m}\sigma_{m}$.

The triangular pattern exhibits the lowest areal pore fraction, yet this ordering does not translate into a proportionally large separation in $\sigma_{m}$. This reduced architecture sensitivity in PETG+CF at low density suggests that the composite’s load transfer and failure are governed not only by macroscopic topology but also by reinforcement-modified local deformation and interface behaviour, which can mask smaller pattern-driven differences under limited replication.

**Figure 24:**
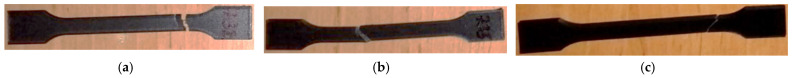
Representative PETG+CF tensile specimens printed with different infill densities: (**a**) 30% (V21A/PETG+CF_732), (**b**) 60% (V22A/PETG+CF_735), and (**c**) 100% (V23A/PETG+CF_739).

**Figure 25:**
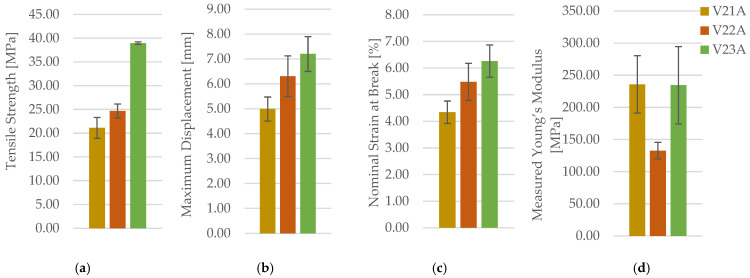
Comparison of tensile properties of representative PETG+CF specimens with different infill densities: (**a**) tensile strength, (**b**) maximum displacement, (**c**) nominal strain at break, and (**d**) measured Young’s modulus.

The effect of infill density in PETG+CF was evaluated for linear infill at 30%, 60%, and 100%. Representative post-test specimens are shown in Figure 24 with representative force–displacement curves in Supplement Materials (Figure S5). Mechanical outcomes are summarized in Table 11 (for clarity and readability, only averaged values (mean ± SD) are reported in the main text, while full individual datasets are provided in the Supplementary Materials Table S5) and property comparisons in Figure 25. One-way ANOVA confirms a highly significant infill-density effect on PETG+CF tensile strength (F(2,6) = 129.25, *p* = 1.17 × 10^−5^). Tukey HSD indicates significant pairwise differences between all density levels (30% vs. 60%: *p* = 1.17 × 10^−2^; 60% vs. 100%: *p* = 1.0 × 10^−4^; 30% vs. 100%: *p* < 1 × 10^−3^).

In contrast to pattern effects at 30%, density exerts a strong and monotonic influence on PETG+CF strength: $\sigma_{m}$ and $F_{max}$ increase substantially from 30% to 60% and further to 100% infill. Deformation measures also increase with density, indicating that in this composite system, densification improves both load capacity and the ability to sustain deformation before fracture under the tested conditions. Statistical summaries (used descriptively due to *n* = 3) confirm that the density effect is large enough to be consistently resolved for $\sigma_{m}$ and $F_{max}$.

**Figure 26:**
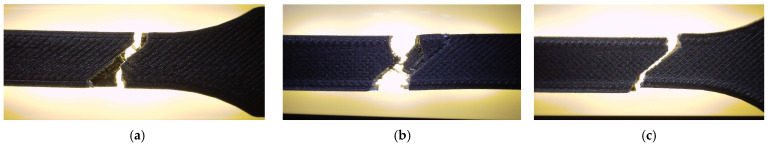
Fracture surfaces of representative PETG+CF specimens after tensile testing: (**a**) V21A/PETG+CF_732—30%; (**b**) V22A/PETG+CF_735—60%; (**c**) V23A/PETG+CF_739—100% infill density.

**Figure 27:**
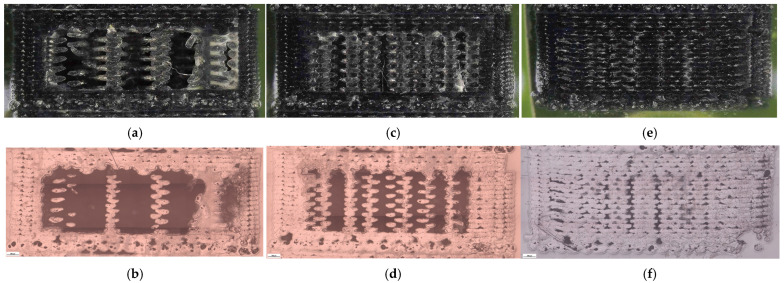
Optical micrographs of representative PETG+CF specimens with different infill densities showing internal morphology: (**a**,**b**) 30% (V21B/PETG+CF_732); (**c**,**d**) 60% (V22B/PETG+CF_735); (**e**,**f**) 100% (V23B/PETG+CF_739).

**Figure 28:**
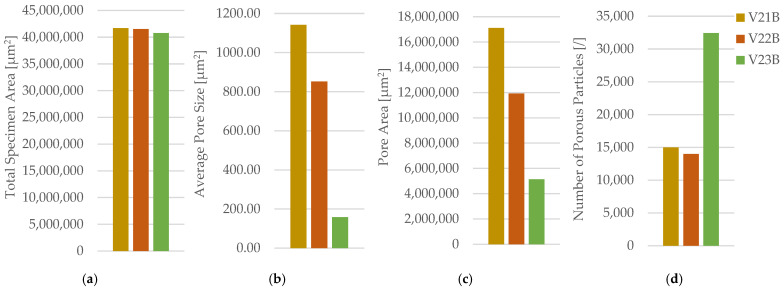
Quantitative microstructural analysis of representative PETG+CF specimens with different infill densities obtained using Fiji software: (**a**) total specimen area, (**b**) average pore size, (**c**) pore area, and (**d**) number of porous particles.

The corresponding fracture surfaces are shown in Figure 26 where higher-density specimens exhibit fracture through a larger effectively solid cross-section. Microstructural cross-sections are presented in Figure 27 with quantitative descriptors in Table 12 and summaries in Figure 28.

The areal pore fraction decreases monotonically with increasing density, total pore area decreases significantly, and the average pore size becomes much smaller at 100% infill while pore count increases, indicating that the residual void population is redistributed into many small features rather than a few large voids. This microstructural transition provides a direct process–structure basis for the strong densification-driven increase in $\sigma_{m}$.

**Figure 29:**
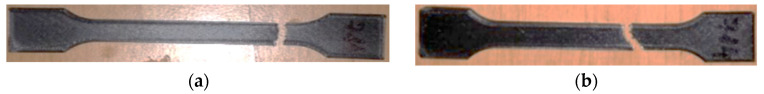
Representative PETG+CF tensile specimens with hexagonal infill (30%): (**a**) unexposed (V19A/PETG+CF_711) and (**b**) exposed to mineral engine oil for 7 days (V24A/PETG+CF_714). Surface softening and darkening are visible after exposure.

**Figure 30:**
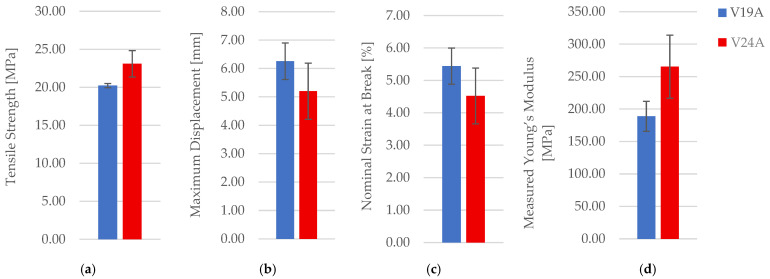
Comparison of tensile properties of representative PETG+CF specimens with hexagonal infill (30%): (**a**) tensile strength, (**b**) maximum displacement, (**c**) nominal strain at break, and (**d**) measured Young’s modulus.

PETG+CF oil exposure was examined for the 30% hexagonal configuration (V19 vs. V24). Representative specimens are shown in Figure 29. Mechanical outcomes are reported in Table 13 (mean ± SD). Representative force–displacement curves (including an oil-exposed specimen) are provided in the Supplementary Materials (Figure S5), and comparisons are shown in Figure 30. A supportive statistical summary of key tensile-strength comparisons is provided in Supplementary Table S6.

**Figure 31:**
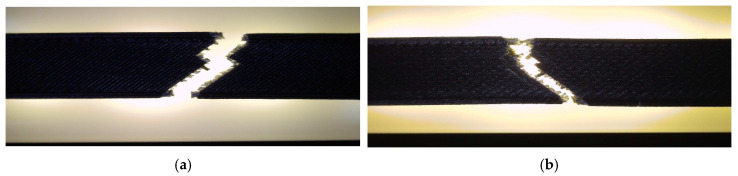
Fracture surfaces of representative PETG+CF specimens after tensile testing: (**a**) unexposed (V19A/PETG+CF_711) and (**b**) 7-day oil-exposed (V24A/PETG+CF_714).

**Figure 32:**
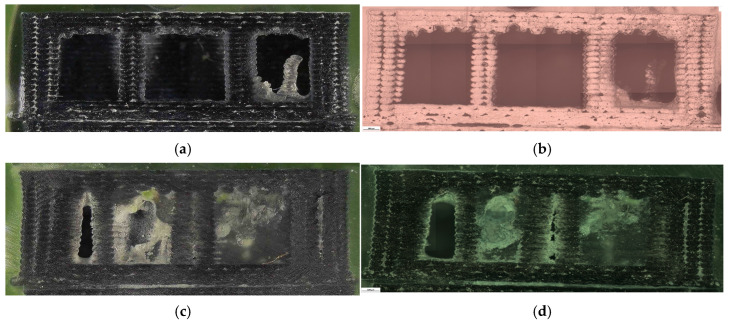
Optical micrographs of unfractured representative PETG+CF specimens with hexagonal infill (30%): (**a**,**b**) unexposed (V19B/PETG+CF_711) and (**c**,**d**) 7-day oil-exposed (V24B/PETG+CF_714).

**Figure 33:**
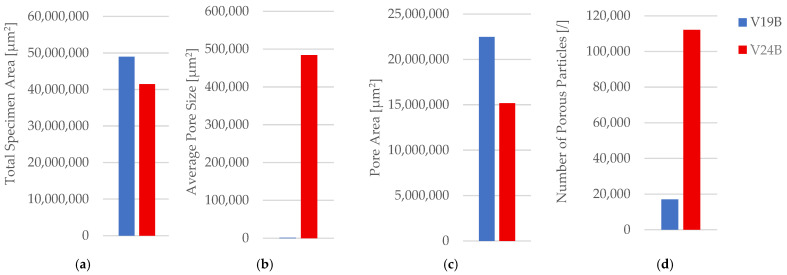
Quantitative microstructural analysis of representative PETG+CF specimens before and after oil exposure: (**a**) total specimen area, (**b**) average pore size, (**c**) pore area, and (**d**) number of porous particles.

In contrast to PETG, PETG+CF shows only modest mean shifts after exposure, and the changes remain within scatter for *n* = 3; thus, the dataset supports the relative stability of the macroscopic tensile response of PETG+CF under the specific lubricant and exposure duration used here. Fracture surfaces in Figure 31 show no clearly distinct fracture mode between unexposed and exposed conditions at the presented magnification. Optical micrographs of unfractured PETG+CF specimens are provided in Figure 32 and pore descriptors are reported in Table 14 with summaries in Figure 33.

After exposure, areal pore fraction decreases, while pore count increases markedly and average pore size decreases, indicating a strong shift towards many small segmented features. Because pore-count metrics are particularly sensitive to thresholding and contrast changes, areal pore fraction and total pore area are treated as the more robust comparative descriptors across exposure conditions.

Across both material systems, the most consistent and largest mechanical effect is produced by densification, which increases $\sigma_{m}$ and $F_{max}$ and is corroborated by a monotonic reduction in areal pore fraction. At full density (linear, 100%), PETG+CF achieves higher $\sigma_{m}$ than neat PETG under matched processing. Under mineral engine oil exposure (hexagonal, 30%), neat PETG exhibits a clear decrease in $\sigma_{m}$ accompanied by an increase in areal pore fraction, whereas PETG+CF shows comparatively small mechanical changes over the same exposure duration, highlighting a material-dependent response.

It should be emphasized that the present lubricant exposure results reflect short-term interaction effects under static immersion conditions. Extrapolation to long-term service performance, cyclic loading, or ageing-controlled degradation mechanisms requires dedicated diffusion, mass uptake, and chemical characterization, which are beyond the scope of this study.

From a comparative application standpoint, the lubricant exposure results demonstrate that service environment constitutes an additional design constraint that interacts strongly with both material selection and internal architecture. While neat PETG exhibits a measurable reduction in tensile strength after oil exposure, the PETG+CF system maintains mechanical stability, indicating that reinforcement mitigates short-term fluid-induced degradation at the structural level.

## 4. Discussion

This section discusses the findings through a process–structure–property lens and translates them into design-relevant guidance for FDM/FFF-printed PETG and PETG+CF. Because tensile testing (ISO 527-2) and porosity segmentation (fixed Fiji-based 2D workflow) were held constant—aligned with our earlier PLA/PLA+CF study [59]—the relative effects of infill topology (30%), infill densification (linear 30–60–100%), and short-term mineral engine-oil exposure can be compared on a consistent basis.

The porosity descriptors reported here are two-dimensional (2D) areal metrics obtained from polished cross-sections and are used for relative comparison under identical imaging and segmentation settings. They do not capture true volumetric porosity, pore connectivity, or through-thickness void networks in anisotropic FDM/FFF structures; such information would require three-dimensional techniques (e.g., X-ray micro-computed tomography) and is identified as future work.

Table 15 consolidates the protocol-controlled comparison across four material systems and highlights the central transferable outcome: densification is the most robust lever for increasing tensile strength, whereas topology effects are most pronounced at low consolidation and are material-specific.

Across the present dataset, infill density is the dominant design lever for strength, supported by a monotonic reduction in areal pore fraction with increasing consolidation. Infill topology primarily shifts the strength–ductility balance at 30% infill, and lubricant exposure acts as an additional service constraint that should be considered during material/architecture selection rather than only during post-print qualification.

This cross-polymer comparison confirms hypothesis H2: increasing infill density increases tensile strength and load capacity through improved load-path continuity and reduced voided area. The observed densification-driven strengthening is consistent with defect-based interpretations widely reported for FDM/FFF structures [22,23,25].

In contrast, topology effects at low density (H1) are strongly material-dependent. It should be noted that topology-related conclusions in the present study are restricted to the low-density regime (30% infill). At higher infill densities, increasing consolidation progressively reduces the influence of raster topology as load transfer becomes dominated by material continuity rather than architectural arrangement. Consequently, pattern-dependent differences observed here are interpreted as low-density design effects and should not be extrapolated directly to highly consolidated structures.

Notably, the optimal pattern at 30% is not universal (PLA: linear; PETG: hexagonal), indicating that infill selection should be treated as material-dependent rather than generic. This difference may be related to polymer-specific inter-road bonding and deformation behaviour (semi-crystalline PLA vs. amorphous PETG) [5,6,14].

The corresponding microstructural evidence supports this interpretation. At 30% infill, different patterns produce distinct defect architectures. Linear infill generates long, aligned inter-road gaps that act as preferential crack-propagation channels (Figure 7e,f), while triangular patterns introduce junction geometries that concentrate stress at sharp angular nodes (Figure 7c,d). Hexagonal infill distributes voids more uniformly within a cellular network (Figure 7a,b), forcing cracks to follow more tortuous paths and enabling stress redistribution. This pattern-dependent defect distribution provides a mechanistic basis for the observed strength–ductility differences and supports H1 beyond purely empirical ranking.

For PETG+CF, the reinforcement modifies this picture. While carbon fibres increase stiffness, the realized reinforcement efficiency is constrained by the same architectural discontinuities that govern load transfer in the neat polymer. At low consolidation, failure can initiate at fibre–matrix debonding sites and interfacial discontinuities that are themselves influenced by the surrounding void architecture [34,39,41]. This coupled interaction between reinforcement, matrix, and printed architecture contributes to increased scatter and less pronounced topology sensitivity in PETG+CF at 30% density.

For PETG+CF, pattern sensitivity at 30% is weaker and scatter is higher, likely because load transfer depends on fibre-related factors (preferred alignment along the extrusion direction, fibre breakage during processing, and local clustering) in addition to architecture-driven discontinuities. These fibre-level descriptors were not quantified here; therefore, reinforcement efficiency is interpreted at the structural level, and detailed fibre orientation/damage analysis is left for future work.

Densification enables the reinforcement benefit: at 100% infill, PETG+CF reaches 38.90 MPa versus 31.35 MPa for neat PETG, consistent with improved fibre–matrix load transfer when porosity and inter-road discontinuities are reduced [34,35,39].

Short-term mineral oil exposure (7 days, hexagonal 30%) provides screening-level evidence of material-dependent sensitivity: neat PETG shows a clear strength reduction, whereas PETG+CF remains within scatter under the same exposure duration.

Because lubricant exposure was evaluated only for one representative low-density configuration (hexagonal, 30%), these results should not be generalized to other topologies or consolidation levels. The intent of this series was to detect early-stage deviations from baseline behaviour under a controlled static immersion condition.

Importantly, no gravimetric mass-uptake measurements, penetration-depth profiling, or chemical characterization (e.g., FTIR) were performed; therefore, the present results do not resolve diffusion kinetics or degradation mechanisms and should not be interpreted as long-term environmental durability.

Within these constraints, the combined mechanical and microstructural findings translate into the following application-oriented guidance, interpreted as screening-level design implications under the present controlled framework:

(i) For static, non-critical components with occasional oil contact, low-density neat PETG may be acceptable with appropriate safety factors;
(ii) For load-bearing applications where repeated or extended oil contact is expected, PETG+CF with moderate-to-high infill density is recommended, with additional long-term qualification advised beyond the present screening protocol;
(iii) For demanding conditions (dynamic loading, impact sensitivity, or strict reliability requirements), high-consolidation PETG+CF is preferred to maximize interfacial integrity and reinforcement effectiveness.

Because n = 3 per condition, statistical tests are used as supportive evidence and conclusions emphasize effect magnitudes, confidence intervals, and consistency across independent indicators (tensile response and porosity descriptors). Young’s modulus values are nominal-area-based and should be interpreted as apparent structural stiffness, particularly at low infill densities.

Overall, the tensile performance of FDM/FFF components emerges from the coupled selection of material, topology (at low density), consolidation degree, and service-fluid contact. The unified workflow used here enables direct, design-relevant comparison across PETG and PETG+CF and supports more reliable parameter selection for lightweight components exposed to lubricants.

Future work may combine this protocol-controlled dataset with orientation-aware modelling and finite-element validation to develop predictive design tools (e.g., [61,62]).

## 5. Conclusions

This study established a controlled process–structure–property dataset for FDM/FFF-printed PETG and PETG+CF by jointly assessing infill topology (30%), infill density (linear 30–60–100%) and 7-day mineral engine oil exposure, while linking tensile performance to consistent microscopy-based porosity descriptors. Within this framework, three conclusions emerge.

First, at lightweight infill (30%), infill topology is a primary design lever for neat PETG and can shift the balance between strength and deformation capacity. Among the tested patterns, the hexagonal architecture provides the most favourable strength–ductility combination, demonstrating that low-density performance cannot be inferred from density alone and must be interpreted through topology-controlled load-path redundancy and interface-driven damage evolution.

Second, for both PETG and PETG+CF, infill density is the dominant and most reliable factor for increasing tensile strength and load capacity. Higher density increases effective structural continuity and strongly reduces pore fraction, supporting a consolidation-driven strengthening mechanism. Reinforcement benefits are most evident at high consolidation, where PETG+CF achieves higher tensile strength than neat PETG under otherwise matched conditions.

Third, mineral engine oil exposure produces a material-dependent response under lightweight architecture. Neat PETG shows a clear reduction in measured tensile strength after 7 days of immersion, indicating that PETG parts intended for lubricant-contact environments—especially at low infill—should be qualified for the intended exposure and designed with appropriate safety margins or with architectural or material modifications. Under the same exposure duration, PETG+CF shows no detrimental change in mean tensile strength, suggesting improved short-term stability for the composite in mineral oil contact within the investigated window.

The main limitation of the present work is the small sample size (*n =* 3 per condition), which constrains statistical generalization and increases sensitivity to specimen-to-specimen variability, which is typical of FDM/FFF due to stochastic defect formation. Future work should therefore priorities higher replicate counts for application-critical configurations, longer and time-resolved exposure protocols (including uptake measurements), and complementary characterization (e.g., SEM fracture surfaces and/or volumetric porosity) to strengthen mechanistic attribution and support design allowable for lubricant-contact service.

Beyond the specific numerical results, this work demonstrates the importance of combining controlled architectural variation with quantitative microstructural descriptors when assessing the mechanical performance of FDM/FFF-printed polymers. The unified process–structure–property framework adopted here provides a transferable methodology that can be extended to other polymers and composite systems, supporting architecture-aware design of lightweight components exposed to service fluids.

Finally, it should be emphasized that the conclusions of this study are restricted to short-term, static tensile response following controlled lubricant exposure. Long-term durability, diffusion-controlled degradation, temperature-dependent effects, and cyclic or fatigue loading behaviour remain outside the scope of the present investigation and represent important directions for future research.

## Figures and Tables

**Table 1:** PETG and PETG+CF filament specifications [60].

Parameter/Material	PETG	PETG+CF
Diameter (mm)	1.75	1.75
Density (g/cm^3^)	1.27–1.28	1.27–1.28
Net filament weight (g)	1000	1000
Water absorption (23 °C/24 h)	<0.2	<0.8
Printing speed (mm/s)	40–60	40–60
Layer height (mm)	0.10–0.20	0.10–0.20
Extrusion temperature (°C)	220–240	230–250
Bed platform temperature (°C)	70–80	50–80
Carbon fibre content (wt%)	0	10

**Table 2:** Mechanical parameters of PETG and PETG+CF filaments [60].

Parameter/Material	PETG	PETG+CF
Tensile Strength (MPa)	40–45	40–43
Young’s modulus (MPa)	1000–1100	2100–2400
Elongation at Break (%)	6.0–8.0	7.5–8.5

**Table 3:** Mechanical parameters of FDM/FFF 3D-printed PETG specimens with different infill geometries.

Case Code	Infill Pattern (30%)	Tensile Strength σ [MPa]	Measured Young’s Modulus E [MPa]	Nominal Strain at Break $\varepsilon_{e}$ [%]	Maximum Displacement Δl [mm]	Maximum Force *F* [N]
V13A	Hexagonal	18.54 ± 0.67	188.97 ± 6.60	7.95 ± 0.65	8.89 ± 0.89	741.69 ± 26.70
V14A	Triangular	14.15 ± 0.70	251.46 ± 50.26	3.79 ± 0.43	4.34 ± 0.51	566.00 ± 28.12
V15A	Linear	16.99 ± 0.52	242.53 ± 34.25	4.69 ± 0.41	5.39 ± 0.47	679.46 ± 20.86

Note (for Table 3 and all mechanical tables below): 95% confidence intervals (CIs) of the mean were calculated using a t-interval with n = 3 (df = 2): $CI=\overset{-}{x}\pm t0.975,2\cdot (s/\surd n).$

**Table 4:** Microstructural parameters of FDM/FFF 3D-printed PETG specimens with different infill geometries.

Case Code	Infill Pattern (30%)	Total Specimen Area [µm^2^]	Number of Porous Particles [/]	Pore Area [µm^2^]	Average Pore Size [µm^2^]
V13B	Hexagonal	42,315,083	9695	16,459,253	1697.71
V14B	Triangular	49,941,774	6987	20,423,996	2923.14
V15B	Linear	50,319,134	6150	22,889,743	3721.91

**Table 5:** Mechanical parameters of FDM/FFF 3D-printed PETG specimens with different infill densities.

Case Code	Infill Density (%)	Tensile Strength σ [MPa]	Measured Young’s Modulus E [MPa]	Nominal Strain at Break $\varepsilon_{e}$ [%]	Maximum Displacement Δl [mm]	Maximum Force *F* [N]
V15A	30%	16.99 ± 0.52	242.53 ± 34.25	4.69 ± 0.41	5.39 ± 0.47	679.46 ± 20.86
V16A	60%	19.10 ± 1.30	328.88 ± 14.54	7.16 ± 1.26	8.24 ± 1.44	764.13 ± 52.15
V17A	100%	31.35 ± 0.33	132.94 ± 26.78	6.43 ± 1.15	7.40 ± 1.32	1253.87 ± 13.30

**Table 6:** Microstructural parameters of FDM/FFF 3D-printed representative PETG specimens with different infill densities.

Case Code	Infill Density (%)	Total Specimen Area [µm^2^]	Number of Porous Particles [/]	Pore Area [µm^2^]	Average Pore Size [µm^2^]
V15B	30%	50,319,134	6150	22,889,743	3721.91
V16B	60%	42,168,952	4285	13,695,646	3196.18
V17B	100%	51,058,246	2729	6,678,236	2447.14

**Table 7:** Mechanical parameters of FDM/FFF 3D-printed PETG specimens with hexagonal infill (30%) before and after mineral oil exposure.

Case Code	Condition	Tensile Strength σ [MPa]	Measured Young’s Modulus E [MPa]	Nominal Strain at Break $\varepsilon_{e}$ [%]	Maximum Displacement Δl [mm]	Maximum Force *F* [N]
V13A	Unexposed	18.54 ± 0.67	188.97 ± 6.63	7.95 ± 0.64	8.89 ± 0.89	741.69 ± 26.72
V18A	7 days exposed	15.42 ± 1.13	286.89 ± 25.55	8.36 ± 0.88	9.62 ± 1.02	616.71 ± 45.15

**Table 8:** Quantitative microstructural parameters of PETG specimens before and after mineral oil exposure.

Case Code	Condition	Total Specimen Area [µm^2^]	Number of Porous Particles [/]	Pore Area [µm^2^]	Average Pore Size [µm^2^]
V13B	Unexposed	42,140,091	39,922	18,972,664	475.24
V18B	7 days exposed	42,135,218	60,168	23,049,106	383.08

**Table 9:** Mechanical parameters of FDM/FFF 3D-printed PETG+CF specimens with different infill geometries (30% density).

Case Code	Infill Pattern (30%)	Tensile Strength σ [MPa]	Measured Young’s Modulus E [MPa]	Nominal Strain at Break $\varepsilon_{e}$ [%]	Maximum Displacement Δl [mm]	Maximum Force *F* [N]
V19A	Hexagonal	20.34 ± 0.28	315.67 ± 23.31	5.15 ± 0.53	5.93 ± 0.61	813.77 ± 11.09
V20A	Triangular	19.21 ± 1.03	222.72 ± 38.08	5.03 ± 1.22	5.79 ± 1.40	768.38 ± 41.11
V21A	Linear	19.98 ± 2.08	257.25 ± 48.69	4.28 ± 0.42	4.92 ± 0.48	799.28 ± 83.73

**Table 10:** Quantitative microstructural parameters of FDM/FFF 3D-printed PETG+CF specimens with different infill geometries.

Case Code	Infill Pattern (30%)	Total Specimen Area [µm^2^]	Number of Porous Particles [/]	Pore Area [µm^2^]	Average Pore Size [µm^2^]
V19B	Hexagonal	48,951,088	17,055	22,464,974	1317.21
V20B	Triangular	41,375,611	19,893	13,669,753	687.16
V21B	Linear	41,709,168	14,981	17,109,895	1142.11

**Table 11:** Mechanical parameters of FDM/FFF 3D-printed PETG+CF specimens with different infill densities (linear infill pattern).

Case Code	Infill Density (%)	Tensile Strength σ [MPa]	Measured Young’s Modulus E [MPa]	Nominal Strain at Break $\varepsilon_{e}$ [%]	Maximum Displacement Δl [mm]	Maximum Force *F* [N]
V21A	30%	19.98 ± 2.08	257.25 ± 48.69	4.28 ± 0.41	4.92 ± 0.48	799.28 ± 83.13
V22A	60%	25.24 ± 1.50	362.40 ± 35.13	5.07 ± 0.64	5.82 ± 0.75	1009.56 ± 15.70
V23A	100%	38.90 ± 0.28	257.51 ± 66.16	6.26 ± 0.61	7.20 ± 0.70	1556.14 ± 11.06

**Table 12:** Quantitative microstructural parameters of FDM/FFF 3D-printed PETG+CF specimens with different infill densities.

Case Code	Infill Density (%)	Total Specimen Area [µm^2^]	Number of Porous Particles [/]	Pore Area [µm^2^]	Average Pore Size [µm^2^]
V21B	30%	41,709,168	14,981	17,109,895	1142.11
V22B	60%	41,518,484	13,987	11,921,091	852.30
V23B	100%	40,768,574	32,442	5,143,342	158.54

**Table 13:** Mechanical parameters of FDM/FFF 3D-printed PETG+CF specimens with hexagonal infill (30%) before and after 7-day oil exposure.

Case Code	Condition	Tensile Strength σ [MPa]	Measured Young’s Modulus E [MPa]	Nominal Strain at Break $\varepsilon_{e}$ [%]	Maximum Displacement Δl [mm]	Maximum Force *F* [N]
V19A	Unexposed	20.34 ± 0.28	315.67 ± 23.31	5.15 ± 0.53	5.93 ± 0.61	813.77 ± 11.09
V24A	7 days exposed	21.30 ± 1.59	302.48 ± 55.40	5.21 ± 0.99	5.99 ± 1.14	851.94 ± 63.70

**Table 14:** Quantitative microstructural parameters of FDM/FFF 3D-printed PETG+CF specimens before and after 7-day oil exposure.

Case Code	Condition	Total Specimen Area [µm^2^]	Number of Porous Particles [/]	Pore Area [µm^2^]	Average Pore Size [µm^2^]
V19B	Unexposed	48,951,088	17,055	22,464,974	1317.21
V24B	7 days exposed	41,460,982	112,133	15,187,770	135.44

**Table 15:** Comparative summary of key outcomes for the present PETG/PETG+CF study and the preceding PLA/PLA+CF study [59] under matched condition definitions (means from *n* = 3 tensile specimens per condition; pore fraction from 2D cross-sections using the fixed Fiji workflow). The linear density series differs between studies (PLA/PLA+CF: 30%, 60%, 90%; PETG/PETG+CF: 30%, 60%, 100%) because the present work includes fully dense (100%) specimens. Oil effects refer to 7-day mineral engine oil immersion for the hexagonal 30% condition. “pp” = percentage points.

Material	Best Tensile Strength at 30% (Patterns) σ [MPa]	Best Nominal Strain at Break at 30% (Patterns) εb [%]	Tensile Strength, Linear Infill σ [MPa]	Pore Fraction, Linear Infill [%]	Oil Effect on σ (Hex 30%)	Oil Effect on εb (Hex 30%)	Oil Effect on Pore Fraction (Hex 30%)
PLA ([59])	lin (31.94)	hex (4.99)	(30%) 31.94	50.17	16.97 → 16.99 (+0.1%)	4.99 → 8.07 (+61.7%)	46.69 → 36.80 (−9.89 pp)
			(60%) 13.53	33.09			
			(90%) 20.34	9.55			
PLA+CF ([59])	hex (18.53)	hex (4.79)	(30%) 16.10	56.51	18.53 → 19.06 (+2.8%)	4.79 → 4.57 (−4.6%)	45.02 → 18.75 (−26.27 pp)
			(60%) 16.10	34.18			
			(90%) 29.03	13.44			
PETG (this study)	hex (18.54)	hex (7.95)	(30%) 16.99	45.49	18.54 → 15.42 (−16.9%)	7.95 → 8.36 (+5.2%)	38.90 → 54.70 (+15.80 pp)
			(60%) 19.10	32.48			
			(100%) 31.35	13.08			
PETG+CF (this study)	hex (20.34)	hex (5.15)	(30%) 19.98	41.02	20.34 → 21.30 (+4.7%)	5.15 → 5.21 (+1.0%)	45.89 → 36.63 (−9.26 pp)
			(60%) 25.24	28.71			
			(100%) 38.90	12.62			

## Data Availability

Data are contained within the article.

## Supplementary Materials

The following supporting information can be downloaded at https://www.mdpi.com/article/10.3390/polym18050654/s1. Table S1: Mechanical parameters of FDM/FFF 3D-printed PETG specimens with different infill geometries; Figure S1: 3D-printed PETG specimens tested: the force–displacement curves. The specimens (a) PETG_411, (b) PETG_421, and (c) PETG_433 are representative samples of case V13A, V14A, and V15A, respectively; Table S2: Individual tensile test results for PETG specimens (linear infill at 30%, 60% and 100% density); Figure S2: 3D-printed PETG specimens tested: the force–displacement curves. The specimens (a) PETG_433, (b) PETG_435, and (c) PETG_438 are representative samples of case V15A, V16A, and V17A, respectively; Table S3: Mechanical parameters of FDM/FFF 3D-printed PETG specimens with hexagonal infill (30%) before and after mineral oil exposure; Figure S3: 3D-printed PETG specimens tested: the force–displacement curves. The specimens (a) PETG_411 and (b) PETG_416 are representative samples of case V13A and V18A, respectively; Table S4: Mechanical parameters of FDM/FFF 3D-printed PETG+CF specimens with different infill geometries (30% density); Figure S4: 3D-printed PETG+CF specimens tested: the force–displacement curves. The specimens (a) PETG+CF_711, (b) PETG+CF_722, and (c) PETG+CF_732 are representative samples of case V19A, V20A, and V21A, respectively; Table S5: Mechanical parameters of FDM/FFF 3D-printed PETG+CF specimens with different infill densities (linear infill pattern); Figure S5: 3D-printed PETG+CF specimens tested: the force–displacement curves. The specimens (a) PETG+CF_711 and (b) PETG+CF_714 are representative samples of case V19A and V24A, respectively. The curves show similar envelopes, consistent with small mean shifts relative to scatter; Table S6: Supportive statistical summary for tensile strength datasets (n = 3 per condition). One-way ANOVA (α = 0.05) with Tukey HSD post-hoc testing was applied for three-level comparisons; Welch’s t-test was applied for two-level comparisons. Given the small sample size, p-values are interpreted as supportive evidence alongside effect magnitudes and CI overlap.

## Abbreviations

The following abbreviations are used in this manuscript:

ABS	Acrylonitrile Butadiene Styrene
ASA	Acrylonitrile Styrene Acrylate
FDM	Fused Deposition Modelling
FFF	Fused Filament Fabrication
PETG	Polyethylene Terephthalate Glycol
PLA	Polylactic Acid
CF	Carbon Fibre
ISO	International Organization for Standardization
SAE	Society of Automotive Engineers
SD	Standard Deviation
CI	Confidence Interval
ANOVA	Analysis of Variance
HSD	Honestly Significant Difference
MAD	Median Absolute Deviation
FOV	Field of View
SEM	Scanning Electron Microscopy
UV-C	Ultraviolet-C
TPU	Thermoplastic Polyurethane
