# Supplementary material for: Mechanical and Microstructural Response of FDM-Printed PETG and PETG+CF to Variable Infill Architecture and Lubricant Exposure
Source: Polymers (Basel). 2026 Mar 7;18(5):654. doi: 10.3390/polym18050654 (PMC12986719; doi:10.3390/polym18050654)
Supplement: Supplementary file 1 [file polymers-18-00654-s001.zip › polymers-4132787-supplementary.pdf]

Table S1. Mechanical parameters of FDM/FFF 3D-printed PETG specimens with different infill geometries.

Case Code	Specimen Code	Tensile Strength σ [MPa]	Measured Young's Modulus E [MPa]	Nominal Strain at Break ε_e [%]	Maximum Displacement Δl [mm]	Maximum Force F [N]
	PETG_411	19.016	186.386	7.321	8.421	760.643
V13A (hexagonal, 30%)	PETG_412	18.832	184.023	8.610	9.910	753.291
	PETG_413	17.778	196.510	7.929	8.325	711.131
Average		*18.542*	*188.973*	*7.953*	*8.885*	*741.688*
St. Dev.		*0.668*	*6.633*	*0.645*	*0.889*	*26.718*
	PETG_421	13.896	227.688	3.441	3.915	555.841
V14A (triangular, 30%)	PETG_422	13.608	309.197	3.650	4.202	544.357
	PETG_423	14.944	217.483	4.263	4.901	597.787
Average		*14.149*	*251.456*	*3.785*	*4.339*	*565.995*
St. Dev.		*0.703*	*50.265*	*0.427*	*0.507*	*28.125*
	PETG_431	16.419	253.119	4.331	4.982	656.788
V15A (linear, 30%)	PETG_432	17.093	270.229	5.134	5.901	683.745
	PETG_433	17.446	204.233	4.592	5.286	697.843
Average		*16.986*	*242.527*	*4.686*	*5.390*	*679.459*
St. Dev.		*0.522*	*34.249*	*0.410*	*0.468*	*20.860*

Note (for Table S3 and all mechanical tables below): 95% confidence intervals (CI) of the mean were calculated using a t-interval with $n = 3$ ($df = 2$): $CI = \bar{x} \pm t0.975{,}2 \cdot (s/\sqrt{n})$.

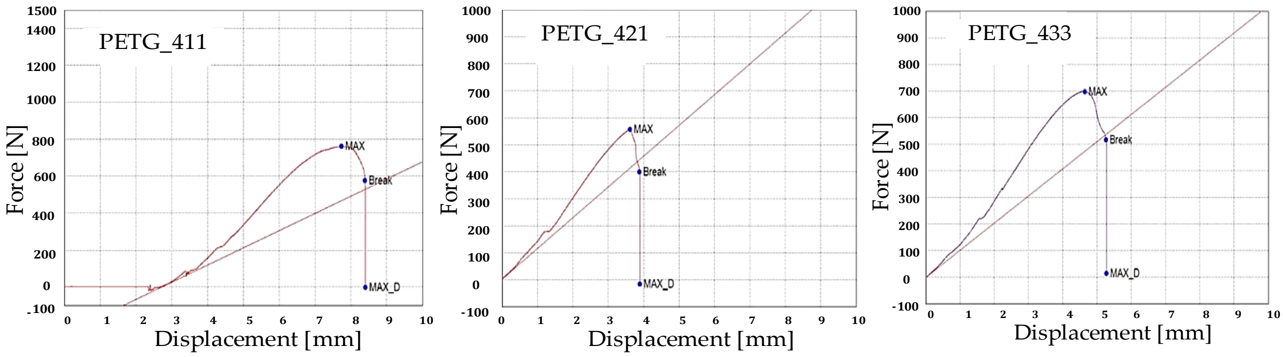

(**a**) (**b**) (**c**)

Figure S1. 3D-printed PETG specimens tested: the force–displacement curves. The specimens (**a**) PETG_411, (**b**) PETG_421, and (**c**) PETG_433 are representative samples of case V13A, V14A, and V15A, respectively.

Table S2. Individual tensile test results for PETG specimens (linear infill at 30%, 60% and 100% density).

Case Code	Specimen Code	Tensile Strength σ [MPa]	Measured Young's Modulus E [MPa]	Nominal Strain at Break ε_e [%]	Maximum Displacement Δl [mm]	Maximum Force F [N]
V15A (linear, 30%)	PETG_431	16.419	253.119	4.331	4.982	656.788
	PETG_432	17.093	270.229	5.134	5.901	683.745
	PETG_433	17.446	204.233	4.592	5.286	697.843
Average		16.986	242.527	4.686	5.390	679.459
St. Dev.		0.522	34.249	0.410	0.468	20.860
V16A (linear, 60%)	PETG_434	19.910	330.066	6.081	7.001	796.429
	PETG_435	19.800	342.788	6.862	7.892	792.003
	PETG_436	17.599	313.780	8.541	9.821	703.963
Average		19.103	328.878	7.161	8.238	764.132
St. Dev.		1.304	14.540	1.257	1.441	52.155
V17A (linear, 100%)	PETG_437	30.972	128.558	7.714	8.867	1238.890
	PETG_438	31.460	108.622	6.082	7.000	1258.410
	PETG_439	31.607	161.634	5.496	6.320	1264.310
Average		*31.346*	*132.938*	*6.431*	*7.396*	*1253.870*
St. Dev.		*0.332*	*26.776*	*1.149*	*1.319*	*13.304*

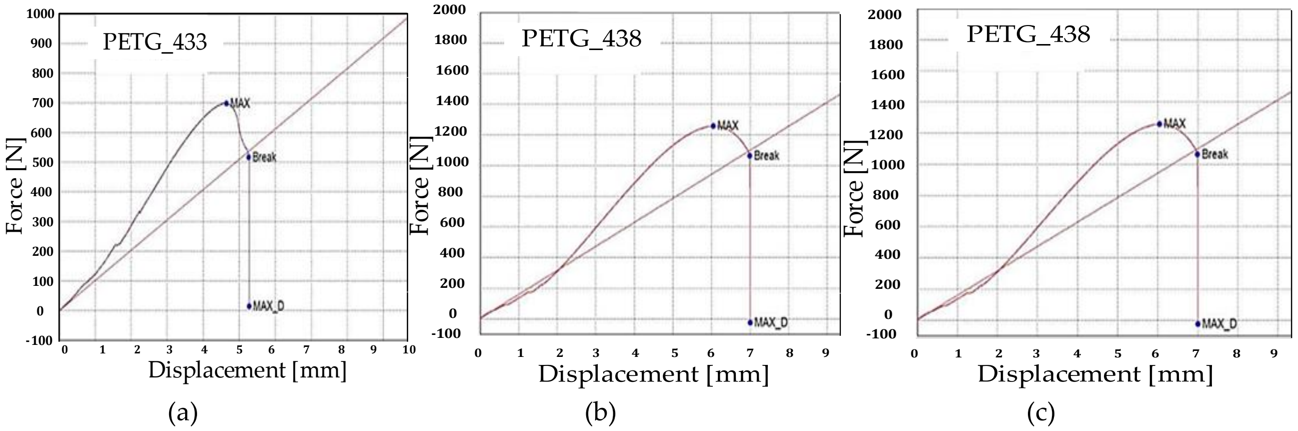

Figure S2. 3D-printed PETG specimens tested: the force–displacement curves. The specimens (**a**) PETG_433, (**b**) PETG_435, and (**c**)PETG_438 are representative samples of case V15A, V16A, and V17A, respectively.

Table S3. Mechanical parameters of FDM/FFF 3D-printed PETG specimens with hexagonal infill (30%) before and after mineral oil exposure.

Case Code	Specimen Code	Tensile Strength σ [MPa]	Measured Young's Modulus E [MPa]	Nominal Strain at Break ε_e [%]	Maximum Displacement Δl [mm]	Maximum Force F [N]
V13A (hexagonal, 30%, unexposed)	PETG_411	19.016	186.386	7.321	8.421	760.643
	PETG_412	18.832	184.023	8.610	9.910	753.291
	PETG_413	17.778	196.510	7.929	8.325	711.131
Average		*18.542*	*188.973*	*7.953*	*8.885*	*741.688*
St. Dev.		*0.668*	*6.633*	*0.645*	*0.889*	*26.718*
V18A (Hexagonal, 30%, 7 days exposed)	PETG_414	14.788	310.963	8.971	10.328	591.532
	PETG_415	14.744	260.086	8.772	10.089	589.760
	PETG_416	16.720	289.632	7.350	8.453	668.836
Average		*15.417*	*286.894*	*8.364*	*9.622*	*616.709*
St. Dev.		*1.128*	*25.549*	*0.884*	*1.020*	*45.152*

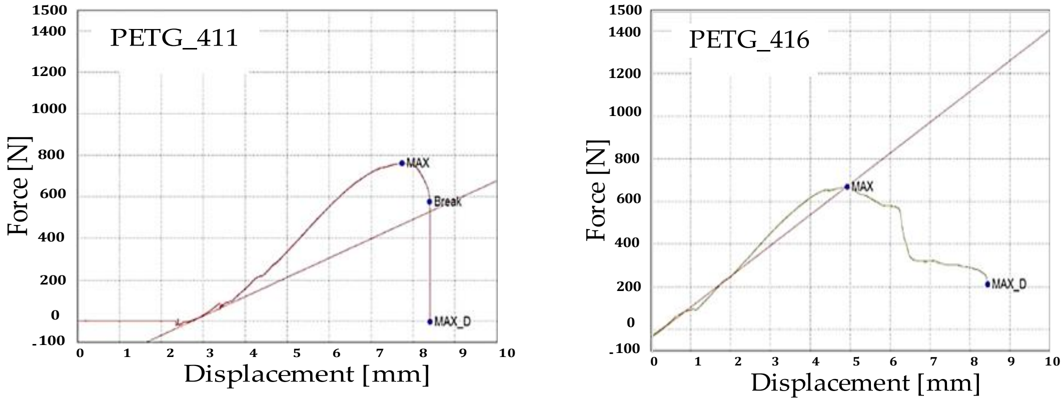

(**a**) (**b**)

Figure S3. 3D-printed PETG specimens tested: the force–displacement curves. The specimens (**a**) PETG_411 and (**b**) PETG_416 are representative samples of case V13A and V18A, respectively.

Table S4. Mechanical parameters of FDM/FFF 3D-printed PETG+CF specimens with different infill geometries (30% density).

Case Code	Specimen Code	Tensile Strength σ [MPa]	Measured Young's Modulus E [MPa]	Nominal Strain at Break ε_e [%]	Maximum Displacement Δl [mm]	Maximum Force F [N]
V19A (hexagonal, 30%)	PETG+CF_711	20.659	327.756	4.541	5.223	826.375
	PETG+CF_712	20.235	288.796	5.440	6.258	809.439
	PETG+CF_713	20.137	330.453	5.478	6.301	805.489
Average		*20.344*	*315.668*	*5.153*	*5.927*	*813.768*
St. Dev.		*0.277*	*23.311*	*0.530*	*0.610*	*11.095*
V20A (triangular, 30%)	PETG+CF_721	19.900	265.200	4.301	4.955	796.032
	PETG+CF_722	19.699	191.641	4.356	5.005	787.966
	PETG+CF_723	18.028	211.311	6.435	7.405	721.137
Average		*19.209*	*222.717*	*5.031*	*5.788*	*768.378*
St. Dev.		*1.028*	*38.083*	*1.216*	*1.400*	*41.110*
V21A (linear, 30%)	PETG+CF_731	17.583	313.116	3.835	4.415	703.351
	PETG+CF_732	21.109	234.759	4.345	4.991	844.375
	PETG+CF_733	21.253	223.862	4.660	5.362	850.121
Average		*19.982*	*257.246*	*4.280*	*4.923*	*799.282*
St. Dev.		*2.079*	*48.691*	*0.416*	*0.477*	*83.129*

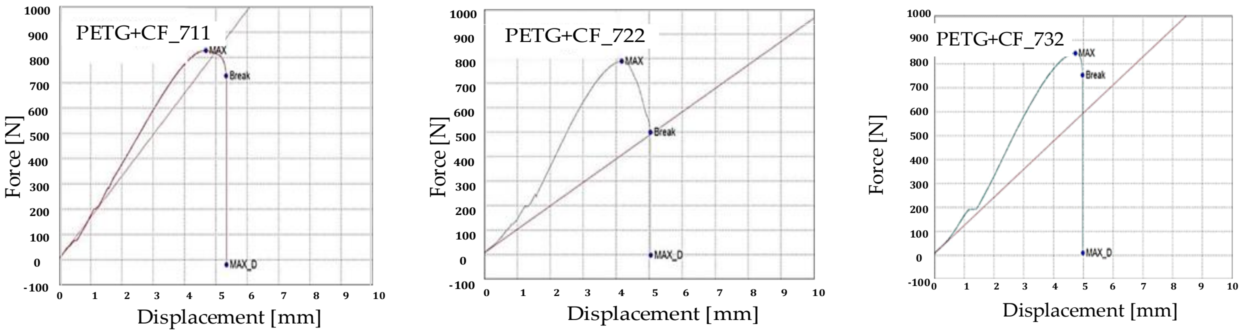

(**a**) (**b**) (**c**)

Figure S4. 3D-printed PETG+CF specimens tested: the force–displacement curves. The specimens (**a**) PETG+CF_711, (**b**) PETG+CF_722, and (**c**) PETG+CF_732 are representative samples of case V19A, V20A, and V21A, respectively.

Table S5. Mechanical parameters of FDM/FFF 3D-printed PETG+CF specimens with different infill densities (linear infill pattern).

Case Code	Specimen Code	Tensile Strength σ [MPa]	Measured Young's Modulus E [MPa]	Nominal Strain at Break ε_e [%]	Maximum Displacement Δl [mm]	Maximum Force F [N]
V21A (linear, 30%)	PETG+CF_731	17.583	313.116	3.835	4.415	703.351
	PETG+CF_732	21.109	234.759	4.345	4.991	844.375
	PETG+CF_733	21.253	223.862	4.660	5.362	850.121
Average		*19.982*	*257.246*	*4.280*	*4.923*	*799.282*
St. Dev.		*2.079*	*48.691*	*0.416*	*0.477*	*83.129*
V22A (linear, 60%)	PETG+CF_734	26.939	353.386	5.391	6.201	1077.590
	PETG+CF_735	24.110	401.163	4.325	4.954	964.435
	PETG+CF_736	24.666	332.650	5.486	6.308	986.640
Average		*25.238*	*362.400*	*5.067*	*5.821*	*1009.555*
St. Dev.		*1.499*	*35.135*	*0.645*	*0.753*	*15.701*
V23A (linear, 100%)	PETG+CF_737	38.608	205.964	5.653	6.501	1544.31
	PETG+CF_738	39.155	332.118	6.869	7.899	1566.21
	PETG+CF_739	38.948	234.440	6.260	7.202	1557.91
Average		*38.904*	*257.507*	*6.261*	*7.201*	*1556.14*
St. Dev.		*0.276*	*66.165*	*0.608*	*0.699*	*11.056*

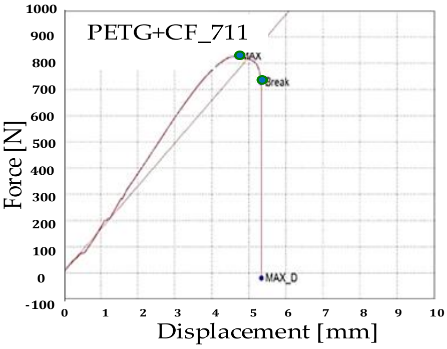

(**a**)

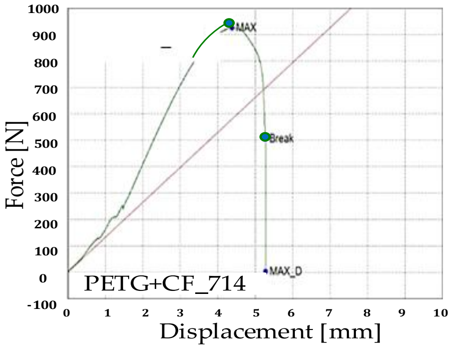

(**b**)

Figure S5. 3D-printed PETG+CF specimens tested: the force–displacement curves. The specimens (**a**) PETG+CF_711 and (**b**) PETG+CF_714 are representative samples of case V19A and V24A, respectively. The curves show similar envelopes, consistent with small mean shifts relative to scatter.

Table S6. Supportive statistical summary for tensile strength datasets (n = 3 per condition). One-way ANOVA (α = 0.05) with Tukey HSD post-hoc testing was applied for three-level comparisons; Welch's t-test was applied for two-level comparisons. Given the small sample size, p-values are interpreted as supportive evidence alongside effect magnitudes and CI overlap.

Material	Dataset / Factor	Test	Test statistic	p-value	Post-hoc / Notes
PETG	Infill pattern (30% density)	One-way ANOVA	F(2,6)=36.81	p=4.28×10−4	Tukey: Triangular vs Hexagonal p=4.0×10−4 (sig.); Triangular vs Linear p=3.8×10−3 (sig.); Hexagonal vs Linear p=5.46×10−2 (n.s.).
PETG	Infill density (linear: 30%, 60%, 100%)	One-way ANOVA	F(2,6)=259.76	p=1.49×10−6	Tukey: 30% vs 60% p=4.74×10−2 (sig.); 60% vs 100% p<1×10−3 (sig.); 30% vs 100% p<1×10−3 (sig.).
PETG	Oil exposure (hexagonal 30%; 7 days)	Welch t-test	t=4.13	p=2.21×10−2	Tensile strength decreases after exposure (supportive significance).
PETG+CF	Infill pattern (30% density)	One-way ANOVA	F(2,6)=0.554	p=6.01×10−1	Tukey: no significant pairwise differences (all n.s.).
PETG+CF	Infill density (linear: 30%, 60%, 100%)	One-way ANOVA	F(2,6)=129.25	p=1.17×10−5	Tukey: 30% vs 60% p=1.17×10−2 (sig.); 60% vs 100% p=1.0×10−4 (sig.); 30% vs 100% p<1×10−3 (sig.).
